# Arsenic trioxide induces macrophage autophagy and atheroprotection by regulating ROS-dependent TFEB nuclear translocation and AKT/mTOR pathway

**DOI:** 10.1038/s41419-020-03357-1

**Published:** 2021-01-18

**Authors:** Shaohong Fang, Xin Wan, Xiaoyi Zou, Song Sun, Xinran Hao, Chenchen Liang, Zhenming Zhang, Fangni Zhang, Bo Sun, Hulun Li, Bo Yu

**Affiliations:** 1 Department of Cardiology, The 2nd Affiliated Hospital of Harbin Medical University, Harbin, Heilongjiang China; 2 The Key Laboratory of Myocardial Ischemia, Harbin Medical University, Ministry of Education, Harbin, Heilongjiang China; 3 Department of Neurobiology, School of Basic Medical Sciences, Harbin Medical University, Harbin, Heilongjiang China

**Keywords:** Macroautophagy, Apoptosis, Atherosclerosis, Experimental models of disease

## Abstract

Inducing autophagy and inhibiting apoptosis may provide a therapeutic treatment for atherosclerosis (AS). For the treatment of progressive AS, arsenic trioxide (ATO) has been used to coat vascular stents. However, the effect of ATO on autophagy of macrophages is still unknown. Therefore, the aims of this study were to characterize the effects and the mechanism of actions of ATO on autophagy in macrophages. Our results showed that ATO-induced activation of autophagy was an earlier event than ATO-induced inhibition of the expression of apoptosis markers in macrophages and foam cells. Nuclear transcription factor EB (TFEB) prevents atherosclerosis by activating macrophage autophagy and promoting lysosomal biogenesis. Here, we report that ATO triggered the nuclear translocation of TFEB, which in turn promoted autophagy and autophagosome-lysosome fusion. Both the latter events were prevented by TFEB knockdown. Moreover, ATO decreased the p-AKT and p-mTOR in the PI3K/AKT/mTOR signaling pathway, thus inducing autophagy. Correspondingly, treatment with the autophagy inhibitor 3-methyladenine (3-MA) abolished the autophagy-inducing effects of ATO. Meanwhile, PI3K inhibitor (LY294002) and mTOR inhibitor (rapamycin) cooperated with ATO to induce autophagy. Furthermore, reactive oxygen species (ROS) were generated in macrophages after treatment with ATO. The ROS scavenger N-acetyl-1-cysteine (NAC) abolished ATO-induced nuclear translocation of TFEB, as well as changes in key molecules of the AKT/mTOR signaling pathway and downstream autophagy. More importantly, ATO promoted autophagy in the aorta of ApoE^−/−^ mice and reduced atherosclerotic lesions in early AS, which were reversed by 3-MA treatment. In summary, our data indicated that ATO promoted ROS induction, which resulted in nuclear translocation of TFEB and inhibition of the PI3K/AKT/mTOR pathway. These actions ultimately promoted macrophage autophagy and reduced atherosclerotic lesions at early stages. These findings may provide a new perspective for the clinical treatment of early-stage atherosclerosis and should be further studied.

## Introduction

Atherosclerosis is a major cause of mortality in the world^1,2^. Studies have established that macrophage apoptosis is crucial for atherosclerotic plaque development^3^. Macrophages recognize and uptake Oxidized low-density lipoprotein (ox-LDL), which then transform into foam cells and eventually undergo apoptosis^4,5^, and thus eventually promote plaque instability^6^.

Autophagy is a mechanism of self-protection closely related to apoptosis^7^, which relies on lysosomes for degradation and recycling of intracellular components to maintain the homeostasis^8^. The locution “autophagy flux” refers to the entire dynamic process of autophagy, including formation of autophagosomes, fusion of autophagosomes with lysosomes, and degradation^9^. More than 30 autophagy-related genes (*Atgs*) and proteins involves in the autophagic process^10,11^. The conversion of LC3I to LC3II is a marker of autophagy activation, whereas the degradation of p62/SQSTM1 is considered an indicator of autophagic protein degradation^12^. Macrophage autophagy plays a protective role in atherosclerosis by enhancing cholesterol outflow^13^. Moreover, macrophage *Atg5* deficiency in Ldlr^−/−^ mice increases apoptosis and promotes plaque necrosis^14^. Thus, therapeutic strategies based on the regulation of apoptosis and autophagy may be suitable for atherosclerosis.

The regulation of autophagy is complex and involves multiple pathways. Sonic dynamic therapy promotes macrophage autophagy by inducing ROS production^15,16^. In addition, phosphoinositide 3 kinase (PI3K)/protein kinase B (AKT)/rapamycin (mTOR) is a classic autophagy pathway^17,18^. Meanwhile, the activation of the ERK1/2 can also induce autophagy^19^. The transcription factor EB (TFEB), a downstream target of mTOR and ERK1/2, is a key regulator of the autophagy-lysosome pathway^20^. TFEB upregulates the expression of nearly two-thirds of autophagy-lysosome pathway-related genes and its overexpression results in strong therapeutic effects in atherosclerosis by rescuing lipid-induced lysosomal dysfunction and downstream sequelae^21^.

Manipulation of macrophage autophagy proved profoundly beneficial in atherosclerosis^22,23^. However, very few autophagic modulators have been reported. Recently, ATO has been shown to reduce neointimal formation and lumen restenosis of the stent surface^24^. In addition, ATO exerts therapeutic effects in acute promyelocytic leukemia by promoting tumor cell apoptosis and autophagic death^25,26^. These reports suggested that ATO regulates apoptosis and autophagy in macrophages, providing a new perspective for the treatment of atherosclerosis.

In this study, we investigated the impact of ATO treatment on macrophage autophagy, as well as the underlying mechanisms. Meanwhile, we evaluated autophagy in atherosclerotic plaque burden in an ATO-treated mouse model of early AS. Our results provide promising perspectives in the use of ATO-induced autophagy as a treatment option for early-stage AS.

## Materials and methods

### Reagents

Arsenic trioxide (ATO, 311383-125G, Sigma, USA), 3-(4,5-dimethylthiazol-2-yl)-2,5-diphenyltetrazolium bromide (MTT, C0009, Beyotime, China), bicolor predye Marker (WJ-102, yazyme, China), 3-methyladenine (3-MA, HY-19312, MCE, China), N-acetyl-1-cysteine (NAC, S0077, Beyotime, China), Chloroquine (CQ, C193834-50mg, Aladdin, China), Lipopolysaccharides (LPS, L2880, Sigma, USA), oxidized low-density lipoprotein (ox-LDL, YB-002, yiyuanBiotech, China), LY294002 (S1105, Selleck, China), Rapamycin (53123-88-9, YESEN, China), Lipofectamine3000 (L3000008, Invitrogen, USA), combine human GM-CSF (300-03, peprotech, USA), mounting-medium (CS703, Dako, China), SYBR Green qPCR Master Mix (HY-K0501, MCE, China), SDS-PAGE (P0015, Beyotime, China), PMSF (ST505, Beyotime, China), Phosphatase inhibitor (Macklin, China), protein extraction reagent nuclear protein extraction kit (R0050, Solarbio, China), hypersensitive ECL chemiluminescencekit (M2301, HaiGene, China), human peripheral blood mononuclear cell isolation kit (TBD, China), HE dye (Right tech, China), modified Oil Red O (G1261, Solarbio, China), DHE fluorescence probe (S0063, Beyotime, China), LysoTracker fluorescent probe (C1046, Beyotime, China), AO-dye (CA1142, Solarbio, China), BSA (Biosharp, China), DAPI (C1002, Beyotime, China), autophagy double-label adenovirus (Hanbio, China).

### Cell isolation and culture

Peripheral blood mononuclear cells were obtained from patients with the approval of the ethics committee of the Second Affiliated Hospital of Harbin Medical University, and written informed consent were obtained from all patients. All of the procedures involving human samples complied with the principles outlined in the Declaration of Helsinki and were approved by Ethics Committee of the Second Affiliated Hospital of Harbin Medical University (KY2017-249, Harbin, China) and performed in accordance with the ethical standards. The cell concentration was 5 × 10^6^/ml, put them in petri dish, added combine human GM-CSF with a concentration of 2000 U/ml in it. Half volume of medium was changed every two days and cells were cultivated for seven days in total. The expression of CD68 was detected by flow cytometry to ensure macrophages were successfully isolated.

Human THP-1 cells, Murine carotid artery endothelial cells (MCEC) and murine carotid artery smooth muscle cells (MCASMC) and Raw264.7 cells were obtained from China Center for Type Culture Collection. The cell lines used in this study were authenticated using short tandem repeat (STR) analysis and regularly tested for mycoplasma. MCEC, MCASMC and Raw264.7 cells were cultured in Dulbecco’s Modified Eagle’s medium (SH30022.01B, HYCLONE, USA) supplemented with 10% fetal bovine serum (0500, ScienCell, USA). Human THP-1 cells and human PBMCs were cultured in 1640 medium (88365, Gibco, USA) supplemented with 10% fetal bovine serum. Cells were free of mycoplasma by PCR analysis and were cultured at 37 degrees Celsius in a humid atmosphere containing 5% CO_2_.

### Animals and experimental protocol

Male ApoE^−/−^ mice with a C57BL/6 background (8-weeks-old) were obtained from Charies River animal center (Beijing, China). All interventions and animal care methods were conducted according to the Guidelines and Policies for Animal Surgery offered by the Animal Ethics Committee of the Second Affiliated Hospital of Harbin Medical University (Harbin, China). The mice were kept in a temperature-controlled facility (temperature: 24 degrees Celsius to 25 degrees Celsius, humidity: 55%) with a 12 h light/12 h dark photoperiod, and food and water were freely available.

ApoE^−/−^ mice were randomized into three groups (4 mice per group) and treated as follows: control (saline, ip.), ATO (2.5 mg/kg /d, ip.), and 3-MA (15 mg/kg /d, ip.) + ATO (combination). Mice were fed a “western diet” for two months, consisting of 78.85% of basic mice maintain feed, 21% of fat and 0.15% of cholesterol. At the start of the second month, mice were fed with intraperitoneal administration of the drugs once every two days for one month, a total of 15 times. The investigator was blinded to the group allocation and assessing the outcome during the experiments.

### Cell viability assay

Cell viability were determined by a MTT Cell Proliferation and Cytotoxicity Assay Kit (Beyotime Biotechnology, Shanghai, China). Raw264.7 cells (5 × 10^3^ cells per well) were seeded into 96-well plates and incubated overnight. After that, Raw264.7 cells were treated with ATO (0, 2.5, 5, 10, 20 μM) or in combination with ox-LDL (84 μg/ml) for 4–48 h. Subsequently, 15 μl of MTT reagents (5 mg/ml) was added. Live cells were counted according to the optical density (OD) of each well which was quantified by an enzyme-linked immunosorbent assay microplate reader at 490 nm. The OD of the results was indicated as the percentage of cell viability in the control group that was set as 100%.

### Real-time quantitative PCR (RT-qPCR)

Total RNAs were extracted using Trizol reagent (ThermoFisher, USA) and reverse-transcribed using the RT Easy II First Strand cDNA Synthesis Kit (04379012001, Roche, Switzerland). Then, cDNA (18 ng) was amplified in a Real-Time PCR Easy (SYBR Green I) (HY-K0501, MCE, China) on Bio-RED Sequence Detection system (Bio-RED, USA). The following primers were used:

*Atg5*, forward 5′-CTTCTGCACTGTCCATCTAAGG-3′ and reverse, 5′-ATCCAGAGTTGCTTGTGATCTT-3′; *Atg7*, forward 5′-GCAGCCAGCAAGCGAAAG-3′ and reverse, 5′-CCGGTCTCTGGTTGAATCTCCTG-3′; Beclin 1, forward 5′-CCCGTGGAATGGAATGAGATTA-3′ and reverse, 5′-CCGTAAGGAACAAGTCGGTATC-3′; LC3, forward 5′-AGAGTGGAAGATGTCCGGCT-3′ and reverse, 5′-CACTTCGGAGATGGGAGTGG-3′; LAMP1, forward 5′-GTTTCTTCATTCTTTACTG-3′ and reverse, 5′-TCTCTACTGTTGTATAGT-3′; β-actin, forward 5′-TAATCTTCGCCTTAATACTT-3′ and reverse, 5′-TAATCTTCGCCTTAATACTT-3′. Gene expression values were normalized against that of β-actin.

### Western blot analysis

After treatments, the cells were lysed in RIPA buffer containing protease and phosphatase inhibitors on ice. The nuclear and cytosolic fractions were obtained by nuclear/cytosol fractionation kit (R0050, Solarbio, China). The concentration of proteins was tested using the bicinchoninic acid (BCA) protein assay. Protein samples (30 μg) were separated by 6%, 10% or 12.5% sodium dodecyl sulfate-polyacrylamide gel electrophoresis and were transferred to 0.22-μm PVDF membranes, followed by blocking for 2 h at room temperature with 5% dried skimmed milk in Tris-buffered saline with 0.05% Tween 20. The membranes were incubated with primary antibodies at 4 degrees Celsius overnight, including LC3 (1:2000, L7543, Sigma, USA), p62 (1:2000, PM045, MBL, Japan), cleaved-caspase-3 (1:2000, ASP175, CST, USA), cleaved-caspase-9 (1:2000, ASP353, CST, USA), GAPDH (1:1000, ta-08, ZSGB-BIO, China), TFEB (1:1000, 13372-1-ap, proteintech, China), DYKDDDDK tag antibody (1:2000, 20543-1-AP, proteintech, China), PCNA (1:10000, 60097-1-lg, proteintech, China), LAMP1 (1:2000, EPR21026, abcam, UK), ATG12 (1:1000, WL03144, Wanleibio, China), PI3K (1:1000, WL02240, Wanleibio, China), p-AKT (1:2000, 4060T, CST, USA), AKT (1:2000, 4691T, CST, USA), p-MTOR (1:2000, AF3308, sangtai-technology, China), mTOR (1:2000, 2983T, CST, USA), p-ERK1/2 (1:2000, 4370T, CST, USA), ERK1/2 (1:2000, 4695T, CST, USA). Subsequently, the membrane was incubated with Horseradish peroxidase (HRP)-conjugated secondary antibodies (1: 8000) for 1 h at room temperature. Immunoreactivity were visualized by chemiluminescence method using ChemiDocTM MP Imaging System (Tanon, China). The protein bands were quantified using a Bio-Rad Chemi EQ densitometer and Bio-Rad Quantity One software (Tanon, China) and normalized to GAPDH or PCNA.

### Analysis of atherosclerosis and plaque histology

Hearts were fixed in OCT for the histological procedures. Sections (8 μm) of the atrioventricular valve region of the heart were collected for haematoxylin–eosin (H&E) staining, and sections (20 μm) were collected for Oil Red O staining. The lipid content, necrotic core area and thickness of fibrous cap were measured on 4 different littermates in each group using Image Pro Plus 6.0.

### Histochemistry and immunohistology analysis

The cross-section samples of the aortic sinus were used for immunofluorescence. The slides were blocked in 10% goat serum diluted with PBS for 30 min and incubated overnight at 4 degrees Celsius with various primary antibodies: LC3 (L7543, Sigma, USA), p62 (PM045, MBL, Japan), CD11b (553312, BD, USA), TFEB (13372-1-ap, proteintech, China), LAMP1 (EPR21026, abcam, UK). After rewarming at room temperature for 10 min, the sections then were incubated with the relevant secondary antibody for another 1 h. The secondary antibodies used were FITC goat anti-rabbit IgG (Invitrogen, USA), TRITC goat anti-rabbit IgG (Invitrogen, USA), FITC goat anti-rat IgG (Invitrogen, USA). The nuclei were stained with 0.5 g/L DAPI for 10 min. Images were obtained using a Confocal microscope (ZEISS LSM 800) and the images were analyzed with Image Pro Plus 6.0.

Cells were seeded onto glass slides. After the indicated treatments, cells were fixed with 4% paraformaldehyde and permeabilized by 0.1% Triton X-100. Subcequently, cells were incubated with primary antibodies and secondary antibody as described in histochemistry, cells fluorescence intensity was observed under Confocal microscope (ZEISS LSM 800) and representative cells were selected and photographed. Quantification analysis was performed using ImageJ.

### Acridine orange (AO) staining

Cells were stained with AO at a concentration of 1 μg/ml for 10 min and then washed with PBS. DAPI was added to label cell nuclei for 1 min at room temperature. Fluorescence intensities were measured by confocal laser scanning microscope (ZEISS LSM 800).

### ROS detection

The ROS generation in cells after ATO treatment was evaluated according to the fluorescence intensity of DHE probe. After ATO treatment, the cells were harvested, washed with PBS, and incubated with 5 μM DHE probe (S0063, Beyotime, China) for 30 min at 37 degrees Celsius in the dark. After rinsing, the fluorescent signals were immediately measured via fluorescence microscope and FACS Verse flow cytometer (BD, American).

### Examination of lysosomal acidification using lysoTracker

The lysosomal acidification was estimated using LysoTracker, following the manufacturer’s instructions (C1046, Beyotime, China). The fluorescence intensity was observed under confocal laser scanning microscope (ZEISS LSM 800) and representative cells were selected and photographed.

### Transfection

Cells were transfected with adenovirus vectors encoding LC3 (HBAD-mRFP-GFP-LC3, HANBIO, China). The transfection efficiency was detected by confocal laser scanning microscope (ZEISS LSM 800) according to the percentage of fluorescence positive cells (>80%). Autophagosomes were represented by the co-localized yellow fluorescence of both GFP and RFP. Due to quenching of GFP signal in acidic compartments, it shows stronger red fluorescence and less co-localization in autolysosomes. LC3 activation was represented by punctate pattern dots in GFP-LC3 transfected cells.

A TFEB mutant with mutation of cysteine to alanine (C211A) and wild type TFEB were constructed by Mijia Biotech (Beijing, China). siTFEB was purchased from GenePharma (Shanghai, China). The target sequences of were listed as followed: Negative control: sense 5′-UUCUCCGAACGUGUCACGUTT-3′; Antisense 5′-ACGUGACACGUUCGGAGAATT-3′; TFEB-mus-324(#1): sense 5′-GCAGGCUGUCAUGCAUUAUTT-3′; Antisense 5′-AUAAUGCAUGACAGCCUGCTT-3′; TFEB-mus-668(#2): sense 5′-CCAUGGCCAUGCUACAUAUTT-3′;

Antisense 5′-AUAUGUAGCAUGGCCAUGGTT-3′; TFEB-mus-1526(#3): sense 5′-CCAAGAAGGAUCUGGACUUTT-3′; Antisense 5′-AAGUCCAGAUCCUUCUUGGTT-3′. Western blots analysis was used to confirmed the efficiency of transfection.

### Statistical analysis

GraphPad Prism 6.0 version software was used for statistical analysis. All experiments were repeated at least three times. Comparisons between two groups were performed using Student’s *t*-test. The variance is similar between the groups that are being statistically compared, data were considered statistically significant if *p* value was less than 0.05.

## Results

### ATO induces autophagy in macrophages

**Fig. 1:**
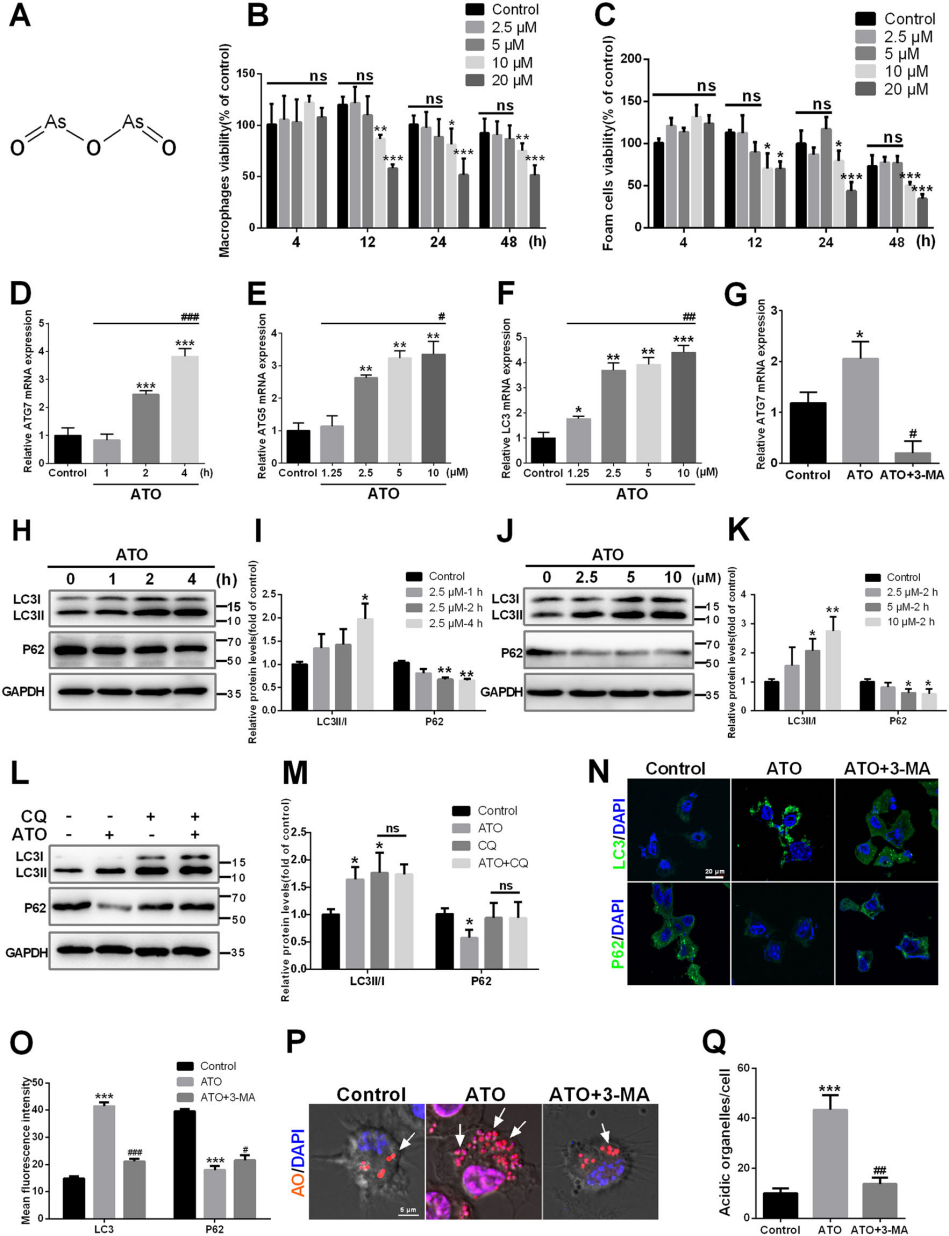
ATO induces autophagy in macrophages. **A** The chemical structure of ATO are shown. **B**, **C** The effects of ATO on the viability of RAW264.7 cells and foam cells with the application of different concentrations (0, 2.5, 5, 10, 20 μM) of ATO for different times (4, 12, 24, 48 h) as indicated. Cell viability was measured by MTT assay. **D** *Atg7* mRNA expression in RAW264.7 cells were analyzed by RT-qPCR after treatment with or without 2.5 μM ATO for 1, 2, 4 h. *p vs. the control group, #*p* vs. the ATO (2.5 μM, 1 h) group. **E**, **F** *Atg5* and LC3 mRNA expression levels in RAW264.7 cells were analyzed by RT-qPCR after treatment with ATO (0, 1.25, 2.5, 5, 10 μM) for 2 h. *p vs. the control group. #*p* vs. the ATO (1.25 μM, 2 h) group. **G** *Atg7* mRNA expression was analyzed by RT-qPCR. *p vs. the control group, #*p* vs. the ATO group. **H**, **I** Western blot analysis of autophagy specific proteins including LC3II/LC3I and p62/SQSTM1 in RAW264.7 cells after treated with ATO (2.5 μM) for 1, 2, or 4 h. **J**, **K** Three specific concentrations of ATO (2.5, 5, 10 μM) were applied for 2 h and protein levels of LC3I, LC3II, and p62 were detected by western blots. **L**, **M** RAW264.7 cells were treated with or without autophagic flux inhibitor CQ (10 μM) for 2 h followed by treated with or without ATO (2.5 μM) for 2 h. Cells were then harvested for western blots to examine p62 and LC3II/I. **N**–**Q** RAW264.7 cells were treated with or without 3-MA (2.5 mM) for 2 h and then treated with or without ATO (2.5 μM) for 2 h. **N**, **O** The imaging of LC3 (green), p62 (green) and DAPI (blue) in RAW264.7 cells were examined by fluorescence microscopy. Scale bars = 20 μm. **P**, **Q** Acidic organelles formation were analyzed by staining with Acridine Orange (AO) and DAPI was added to label nuclei (blue). Scale bars = 5 μm. Data were expressed as mean ± SD from 3 independent experiments. *p < 0.05, **p < 0.01, ***p < 0.001, ^#^*p* < 0.05, ^##^*p* < 0.01, ^###^*p* < 0.001, and ns means non-significant.

Previous studies have tested the effects of ATO in various cell lines, and reported a wide range of effects, from cell survival to cell death^27–29^. Figure 1A shows the chemical structure of ATO. Cell viability of macrophage and foam cells were tested when treated with various drug concentrations and exposure times. Cell viability was decreased after ATO (≥10 μM) exposure for 12–48 h. At concentrations ≤5 μM, however, cell viability was not affected before 48 h (Fig. 1B, C). Therefore, ATO doses ≤5 μM were used in subsequent experiments. To explore the impact of ATO on macrophage autophagy, we first examined the expression of several autophagy-related genes. Macrophages treated with 2.5 μM ATO exhibited increased levels of *Atg7* mRNA in a time-dependent manner, compared to controls (Fig. 1D). Moreover, cell exposure to different ATO concentrations (1.25 μM–10 μM) for 2 h resulted in dose-dependent increases in the levels of *Atg5* and LC3 mRNAs (Fig. 1E, F). The conversion of LC3I to LC3II and the degradation of p62, specific markers of activation and complication of autophagy, were also examined^30^. Treatment of macrophages with 2.5 μM ATO increased the LC3II/LC3I ratio and decreased p62 in a time-dependent manner (Fig. 1H, I). The latter effects were also dose-dependent (Fig. 1J, K). Moreover, compared to the control group, CQ increased LC3II/LC3I but blocked the degradation of p62, while ATO increased LC3II/LC3I and promoted degradation of p62, suggesting that ATO promotes the completion of the autophagic flux (Fig. 1L, M). Moreover, the autophagy inhibitor 3-MA, suppressed *Atg7* mRNA expression (Fig. 1G), LC3II accumulation, and p62 degradation (Fig. 1N, O) induced by 2.5 μM ATO. Consistently, acridine orange (AO) staining showed that 2.5 μM ATO increased red signal (acidic organelles). On the other hand, 3-MA abolished ATO-induced organelle acidification (Fig. 1P, Q). These data demonstrated that ATO promotes the formation of autophagosomes and the autophagic flux through lysosomes.

### ATO induce autophagy earlier than inhibiting the expression of apoptosis markers in macrophages and foam cells

**Fig. 2:**
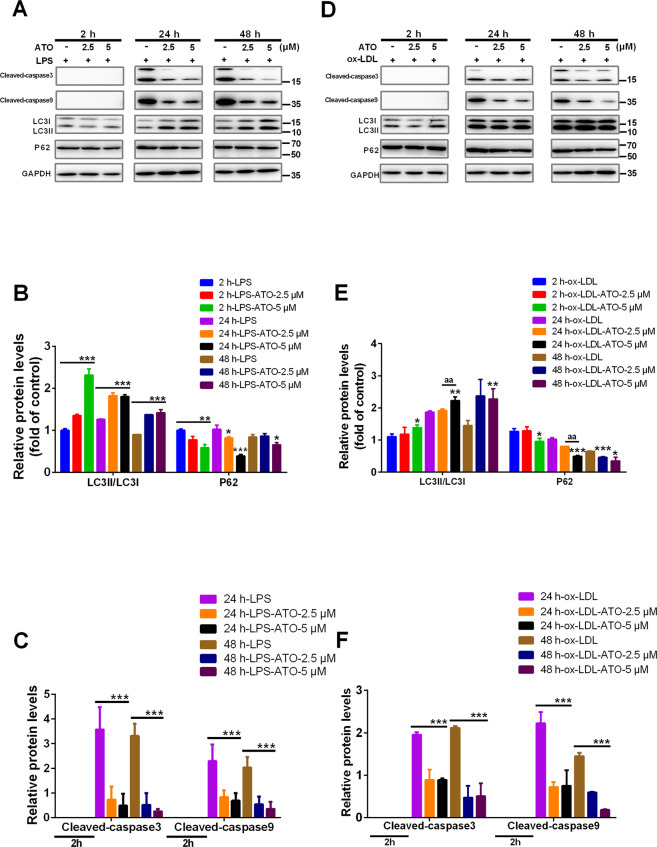
ATO induce autophagy earlier than inhibiting the expression of apoptosis markers in macrophages and foam cells. **A**–**C** WB analysis of autophagy and apoptosis specific proteins including LC3, P62, cleaved-caspase-3 and cleaved-caspase-9 in RAW264.7 cells after treatment with 100 ng/ml LPS for 6 h, followed by treatment with ATO (0, 2.5, 5 μM) for 2, 24, or 48 h. *p < 0.05, **p < 0.01, ***p < 0.001 vs. LPS group. **D**–**F** WB analysis of autophagy and apoptosis specific proteins including LC3, P62, cleaved-caspase-3 and cleaved-caspase-9 in RAW264.7 cells after treated with 84 μg/ml ox-LDL and ATO (0, 2.5, 5 μM) for 2, 24, 48 h. Data were expressed as mean ± SD from 3 independent experiments. *p < 0.05, **p < 0.01, ***p < 0.001 vs. ox-LDL group, aa*p* < 0.01 vs. Specified group, and ns means non-significant.

Autophagy and apoptosis have been reported to be activated at different stages^31,32^. Cleaved caspase-3 (cl-caspase-3) and cleaved caspase-9 (cl-caspase-3) are reliable markers for apoptosis^33^. To explore the dynamics effect of ATO on autophagy and apoptosis, we examined the expression of LC3II, p62, cl-caspase-3, and cl-caspase-9. Lipopolysaccharides (LPS, 100 ng/ml) was employed to trigger apoptosis in macrophages^34^. After macrophage stimulation with ATO (2.5, 5 μM) for 2–48 h, LC3II/LC3I was increased but p62 was decreased, compared with that in the LPS group (Fig. 2A, B). Interestingly, cl-caspase-3 and cl-caspase-9 were expressed in macrophages at 24 and 48 h, but not at 2 h. After stimulation with ATO (2.5, 5 μM) for 24 and 48 h, the expression of cl-caspase-3 and cl-caspase-9 was reduced in macrophages compared with the LPS group (Fig. 2A, C). These results indicated that ATO induced macrophage autophagy at 2 h but inhibited the expression of apoptosis markers at 24 h.

Apoptosis caused by ox-LDL overload in foam cells is a sign of atherosclerosis. To mimic and explore the dynamic effects of ATO on autophagy and apoptosis in this process, we used 84 μg/ml ox-LDL to induce foam cells formation in vitro (Fig. 2D–F). Although after 2 h of treatment, neither caspase-3 nor caspase-9 were expressed (Fig. 2D, F), macrophages treated with ox-LDL for 24 and 48 h had detectable levels of both caspases. Meanwhile, ATO (2.5, 5 μM) reduced the expression of cl-caspase-3 and cl-caspase-9 (Fig. 2D, F). In addition, exposure to ATO (2.5, 5 μM) for 2–48 h increased the LC3II/LC3I ratio and reduced p62 compared to ox-LDL-treated macrophages (Fig. 2D, E). These results indicated that ATO induces autophagy in foam cells at 2 h, much earlier compared to its inhibition of apoptosis markers at 24 h.

### ROS contribute to ATO-induced autophagy in macrophages

**Fig. 3:**
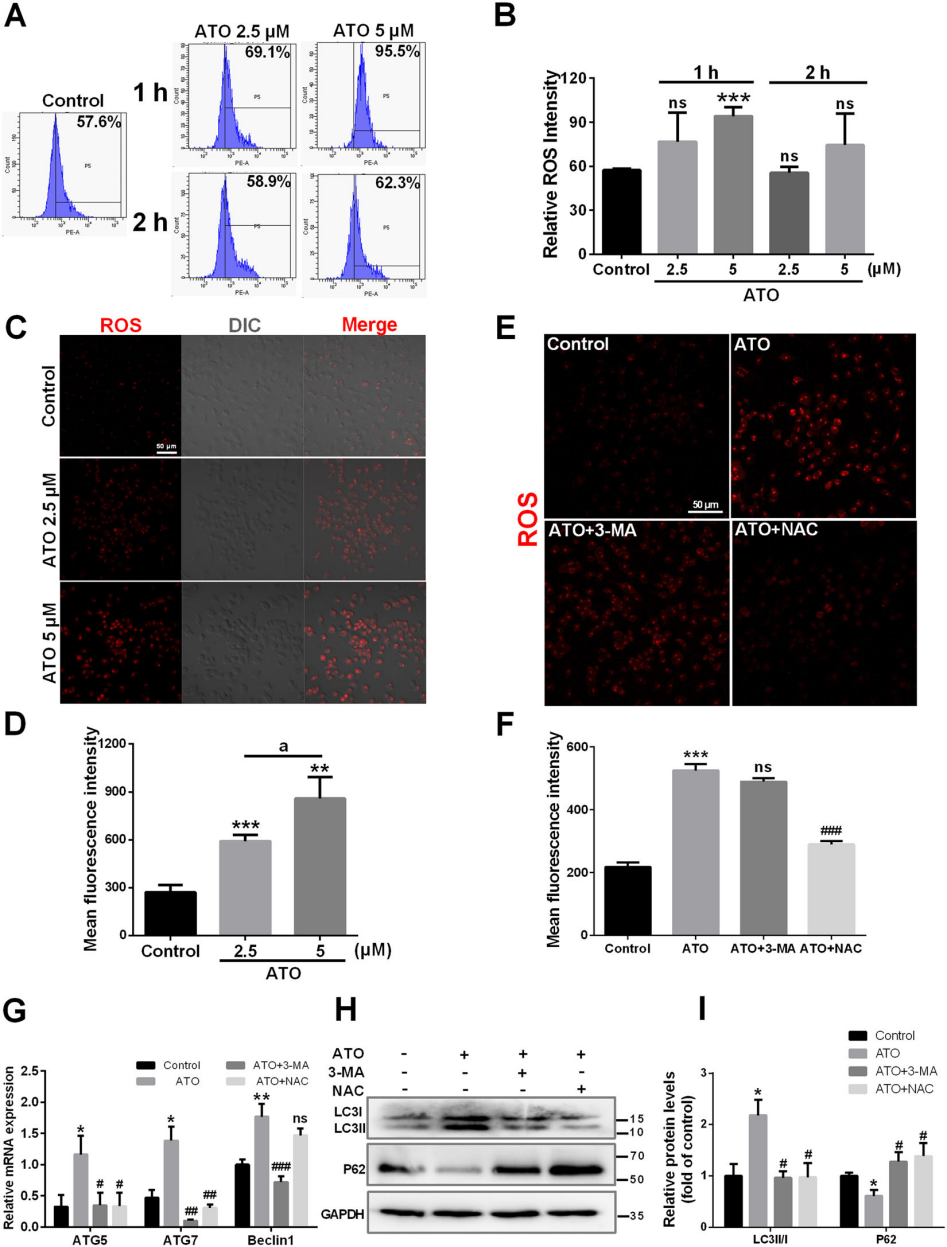
ROS contribute to ATO-induced autophagy in macrophages. **A**, **B** RAW264.7 cells were treated with two concentrations of ATO (2.5, 5 μM) for 1 h and 2 h. The relative fluorescence intensity for ROS generation was analyzed by flow cytometry. **C**, **D** Fluorescence microscopy images of ROS generation in RAW264.7 cells after treated with ATO (2.5, 5 μM) for 1 h. Scale bars=50 μm. a*p* < 0.05 vs. specified group. **E**–**I** RAW264.7 cells were treated with or without 3-MA(2.5 mM) or NAC (5 mM) followed by ATO (2.5 μM) for 2 h. **E**, **F** Fluorescence microscopy images of ROS generation in RAW264.7 cells in the presence of NAC or 3-MA following ATO treatment. Scale bars = 50 μm. **G** RT-qPCR analysis of ROS effect on autophagy via detecting specific autophagy genes including *Atg5*, *Atg7* and Beclin 1 following incubate treatment. *p vs. the Control group, #*p* vs. the ATO group. **H**, **I** WB analysis of ROS effect on autophagy via detecting autopahgy specific proteins LC3 and P62 following incubate treatments. Data were expressed as mean ± SD from 3 independent experiments. *p < 0.05, **p < 0.01, ***p < 0.001, and ns means non-significant.

It has been shown that ROS promote the activation of autophagy in macrophages^35^. Here, treatment for 1 h with ATO (2.5, 5 μM) induced a dose-dependent increase in ROS production in macrophages (Fig. 3A–F). This effect was abolished at 2 h (Fig. 3A, B). In addition, NAC abolished ROS production, while 3-MA did not show this effect (Fig. 3E, F). We next assessed whether ROS were involved in ATO-induced autophagy. ATO (2.5 μM) increased *Atg5*, *Atg7*, and Beclin 1 mRNA levels in macrophages, compared to control cells (Fig. 3G), as well as the LC3II/LC3I ratio and p62 degradation (Fig. 3H, I). Furthermore, 3-MA treatment abolished all ATO-induced changes in autophagy-related genes and proteins (Fig. 3G, I). However, NAC prevented these effects incompletely (Fig. 3G, I). These results demonstrated that increased ROS production is one of the upstream events contributing to ATO-induced activation of macrophage autophagy.

### ATO stimulates ROS-dependent nuclear translocation of TFEB in macrophages

**Fig. 4:**
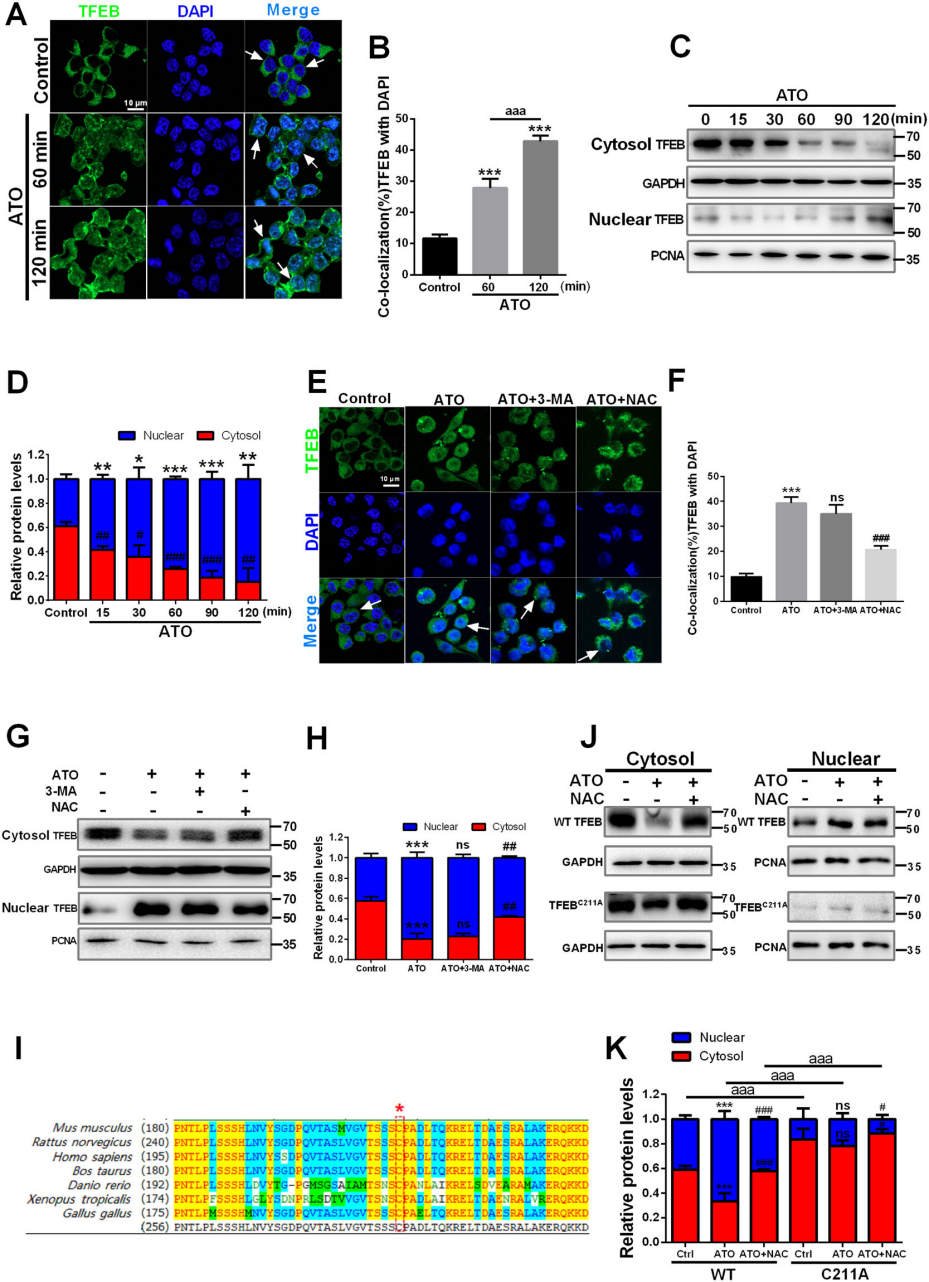
ATO stimulates ROS-dependent nuclear translocation of TFEB in macrophages. **A**, **B** RAW264.7 cells were treated with or without 2.5 μM ATO for 60 min and 120 min. After immunostaining with TFEB (green) and DAPI (blue), cells were examined by fluorescence microscopy. Scale bars = 10 μm. The colocalization of TFEB and DAPI was calculated and analyzed (right panel) aaa*p* < 0.001 vs. Specified group. **C**, **D** WB analysis of subcellular distribution of TFEB in RAW264.7 cells between cytosol and nucleus after treated with 2.5 μM ATO for indicated time (15, 30, 60, 90, 120 min). The data are quantified in the panel d. *p vs. TFEB relactive expression in nuclear of control group, #*p* vs. TFEB relactive expression in cytoplasm of control group. **E**, **F** Immunofluorescence analysis of the effects of 3-MA (2.5 mM, 2 h) and NAC (5 mM, 2 h) on TFEB nucleus translocation in RAW264.7 cells following ATO (2.5 μM, 2 h) treatment. Scale bars=10 μm. **G**, **H** The effects of 3-MA (2.5 mM, 2 h) and NAC (5 mM, 2 h) on TFEB nucleus translocation in RAW264.7 cells following ATO (2.5 μM, 2 h) treatment were detected by western blots. **I** Protein sequence alignment of TFEB homologs in different species. The asterisk indicates the evolutionarily conserved cysteine residue. **J**, **K** RAW264.7 cells were transfected with WT or mutant TFEB C211A. After 24 h, the cells were treated with ATO (2.5 μM) for 15 min, which was pre-treated with or without NAC (5 mM) for 2 h. Then, the cell lysates were subjected to subcellular fractionation. The separated fractions were analyzed by immunoblotting with antibodies. *p vs. TFEB relative expression of Control group, #*p* vs. TFEB relative expression of ATO group, aaa*p* < 0.001 vs. Specified group. Data were expressed as mean ± SD from 3 independent experiments. *p < 0.05, **p < 0.01, ***p < 0.001, and ns means non-significant.

TFEB is a key regulator of lipid clearance in atherosclerosis^36^. Normally, TFEB is phosphorylated by mTOR in the cytosol. When dephosphorylated, TFEB translocates into the nucleus to promote autophagosomal and lysosomal gene expression, thereby enhancing autophagy and lysosomal biogenesis^37,38^. However, ROS generation can also induce a rapid translocation of TFEB by oxidizing its sole cysteine residue^39^. Firstly, to explore the potential role of TFEB in ATO-induced autophagy, we examined the intracellular localization of TFEB following treatment with 2.5 μM ATO (Fig. 4). A 15-min treatment with 2.5 μM ATO promoted the nuclear translocation and accumulation of TFEB, while decreasing its cytosolic fraction, as assessed by immunofluorescence and western blot. These effects were more evident after longer treatments and up to 120 min (Fig. 4A–D). Furthermore, to discriminate the relationship between ROS generation, autophagy activation and ATO-triggered TFEB nuclear translocation, we tested the effects of NAC and 3-MA on them. After treatment with 2.5 μM ATO, TFEB was clearly localized into the nucleus, with or without 3-MA, as determined by both immunofluorescence (Fig. 4E, F) and western blot analyses (Fig. 4G, H). However, all the described ATO-induced changes were reversed by NAC (Fig. 4E–H). These results demonstrated that the nuclear translocation of TFEB triggered by ATO is ROS-dependent and is an upstream event in ATO-induced autophagic activation.

To this end, we analyzed the protein sequence alignment of TFEB homologs in different species and identified the sole cysteine residue in mice TFEB, cysteine 211, that is highly conserved among various species (Fig. 4I). We speculated that the promotion of the oxidation of this cysteine residue under ATO treatment is responsible for the rapid nuclear translocation of TFEB. To test this, we constructed a TFEB mutant with mutation of cysteine to alanine (C211A). Under ATO treatment within 15 min, wild-type TFEB translocated into the nucleus rapidly. In contrast, TFEB C211A failed to translocate into the nucleus within the same time (Fig. 4J, K). These results indicate that, ATO promotes the oxidation of C211 to cause a rapid nuclear transport of TFEB.

### ATO promotes TFEB-dependent lysosome biosynthesis and the fusion between autophagosomes and lysosomes in macrophages

**Fig. 5:**
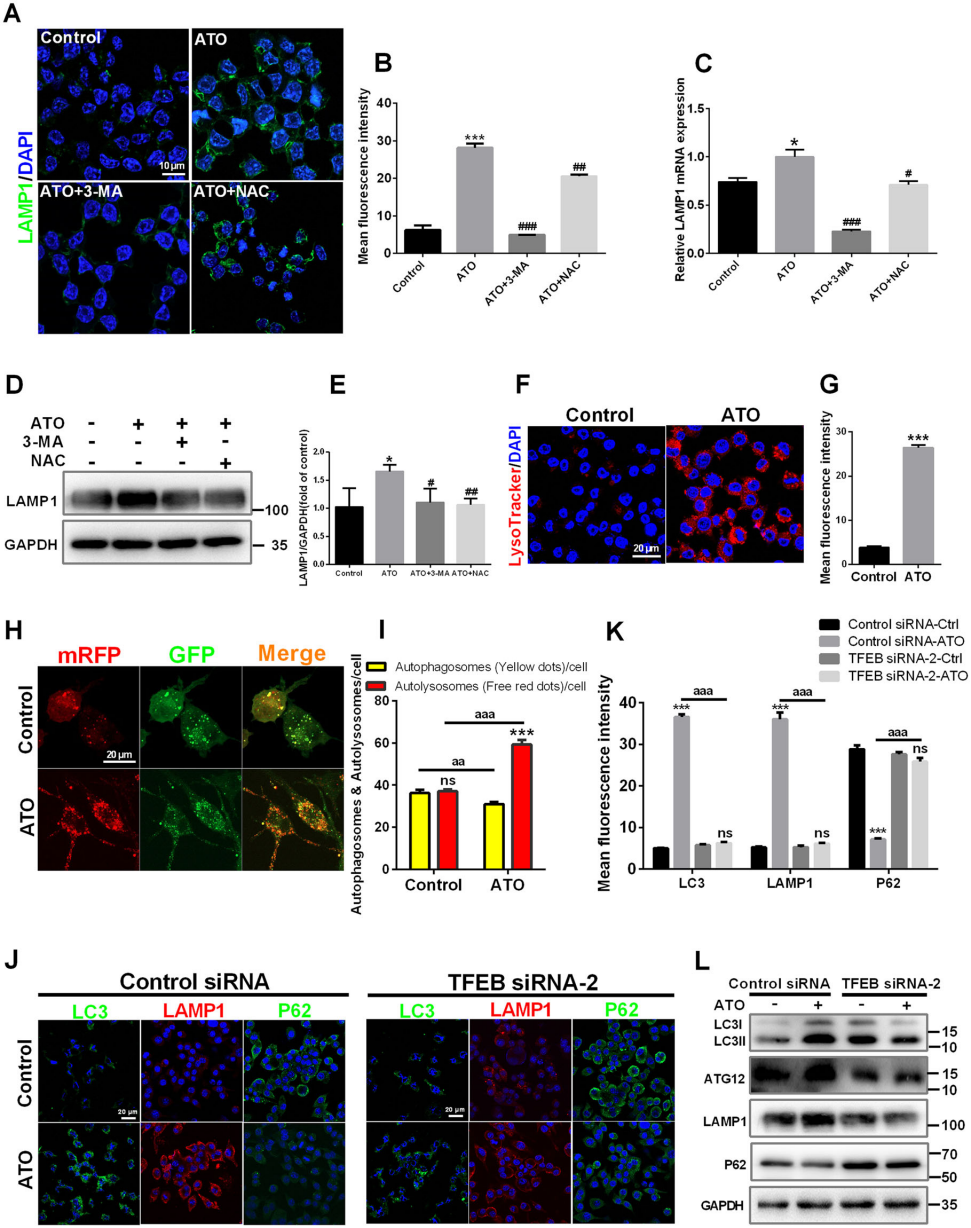
ATO promotes TFEB-dependent lysosome biosynthesis and the fusion between autophagosomes and lysosomes in macrophages. **A–E** RAW264.7 cells were treated with 3-MA (2.5 mM) or NAC (5 mM) for 2 h following ATO treatment (2.5 μM, 2 h). Then LAMP1 level in treated cells were analyzed by immunostaining (**a**, **b**), RT-qPCR (**c**), and western blots (**D**, **E**). Scale bars = 10 μm. **F**, **G** LysoTracker staining in RAW264.7 cells treated with or without 2.5 μM ATO for 2 h. Scale bars = 20 μm. **H**, **I** RAW264.7 cells were transfected with GFPmRFP-LC3 for 48 h and then treated with ATO (2.5 μM) for 2 h. The numbers of acidified autophagosomes (GFP^−^RFP^+^) versus neutral autophagosomes(GFP^+^RFP^+^) per cell were examined and quantified by confocal microscopy. Scale bars=20 μm. ***p < 0.001 vs. autophagosomes of ATO group. aa*p* < 0.01, aaa*p* < 0.001 vs. specified group. **J**–**L** RAW264.7 cells were transfected with scrambled or TFEB siRNA for 48 h, and then treated with ATO (2.5 μM) for 2 h. **J**, **K** Immunofluorescence analysis of TFEB effect on lysosomal and autophagy dysfunction in RAW264.7 cells as assessed LC3 (green), LAMP1 (red), p62 (green) and DAPI (blue) following indicated treatments. Scale bars = 20 μm. aaa*p* < 0.001 vs. specified group. **L** WB analysis of TFEB effect on lysosomal and autophagy dysfunction in RAW264.7 cells as assessed autophagy specific proteins following indicated treatments. Data were expressed as mean ± SD from 3 independent experiments. *p < 0.05, **p < 0.01, ***p < 0.001, and ns means non-significant.

During the final stages of autophagy, autophagosomes fuse with lysosomes and are subsequently degraded, which is necessary for lysosomal activation^40^. First, lysosome biosynthesis was examined. ATO (2.5 μM) increased LAMP1 expression in macrophages as demonstrated by immunofluorescence, qPCR, and western blot analysis (Fig. 5A–E). Treatment with 3-MA abolished ATO-induced LAMP1 upregulation, while NAC abolished this phenomenon incompletely (Fig. 5A–E). Moreover, in ATO-treated macrophages, the fluorescence of LysoTracker-stained cells was increased, suggesting that ATO enhanced lysosomal acidification (Fig. 5F, G). These results indicated that ATO increased the number and enhanced the function of lysosomes, and that ROS was one of the upstreams of these effects. Moreover, we used mRFP-GFP tandem fluorescent‑tagged LC3II to examine autophagosome−lysosome fusion. Treatment with 2.5 μM ATO resulted in an increase in RFP-only puncta, suggesting that ATO promoted autophagosome−lysosome fusion (Fig. 5H, I). Next, TFEB siRNA was used to assess the contribution of TFEB to ATO-induced autophagic signaling. TFEB siRNA-2 knocked down TFEB expression effectively (Supplementary Fig. S1d). Therefore, TFEB siRNA-2 and control siRNA were used in subsequent experiments (Fig. 5J–L). As expected, 2.5 μM ATO combined with control siRNA increased LC3II/LC3I, *Atg12* and LAMP1 expression, but reduced p62, compared to cells transfected with control siRNA alone (Fig. 5L). However, all these effects were prevented by TFEB siRNA-2 (Fig. 5J–L). Interestingly, treatment with 2.5 μM ATO caused an increase in p-ERK1/2 expression, but TFEB siRNA-2 did not affect this change (Supplementary Fig. S1e). In summary, ATO promoted TFEB-dependent lysosome biosynthesis, as well as the fusion between autophagosomes and lysosomes in macrophages.

### ATO induces autophagy in macrophages by inhibiting the PI3K/AKT/mTOR pathway

**Fig. 6:**
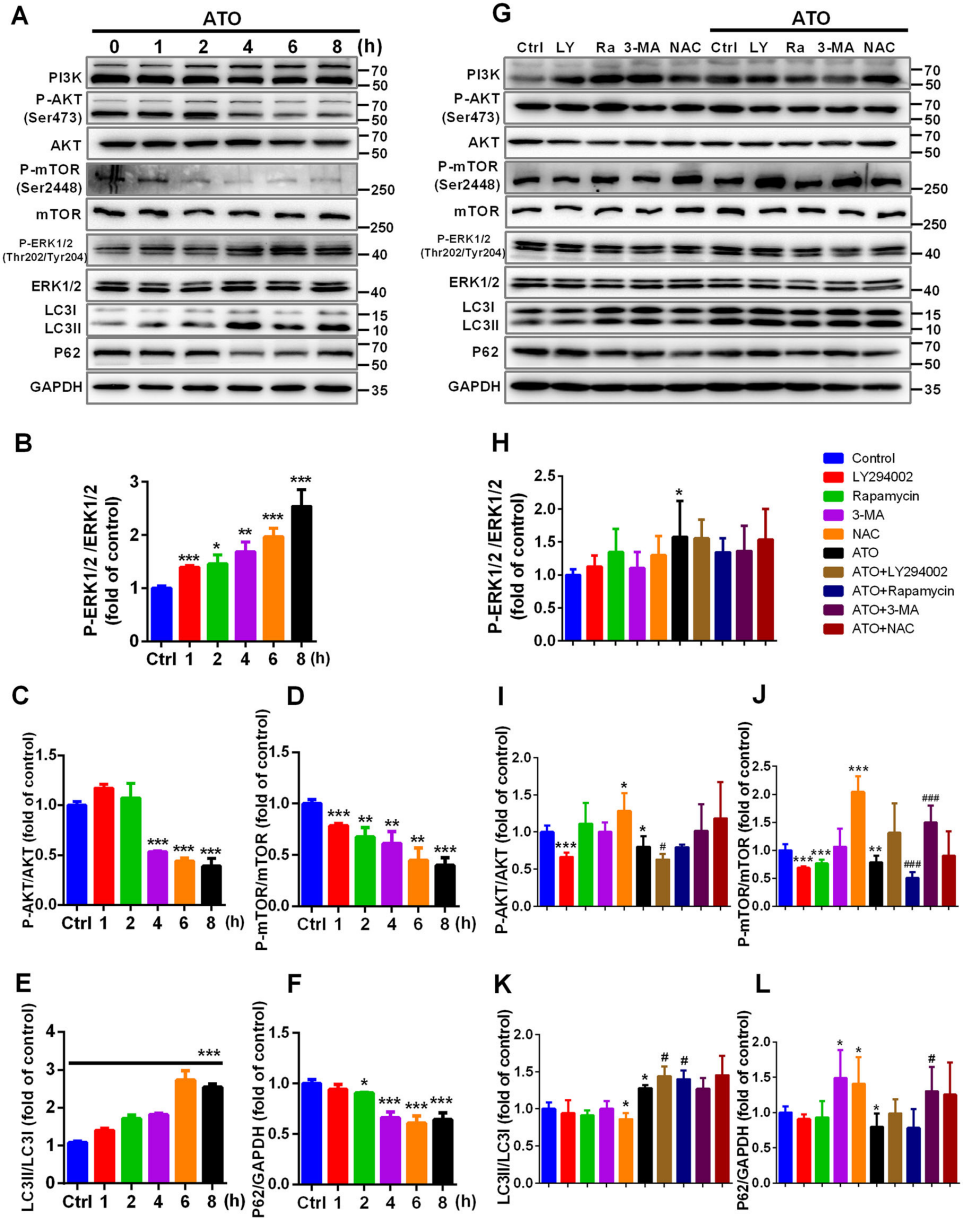
ATO induces autophagy in macrophages by inhibiting the PI3K/AKT/mTOR pathway. **A**–**F** WB analysis of autophagy signaling pathway proteins, LC3II/LC3I and p62 in RAW264.7 cells treated with 2.5 μM ATO for 1, 2, 4, 6, 8 h. **G**–**L** WB analysis of autophagy signaling pathway proteins, LC3II/LC3I and p62 in RAW264.7 cells pre-treated with LY294002 (10 nM), rapamycin (100 nM), 3-MA (2.5 mM), or NAC (5 mM) for 2 h respectively, and then treated with or without 2.5 μM ATO for 2 h. *p vs. Control, #*p* vs. ATO. Data were expressed as mean ± SD from 3 independent-experiments. *p < 0.05, **p < 0.01, ***p < 0.001, ^#^*p* < 0.05, ^###^*p* < 0.001, and ns means non-significant.

ERK1/2 and AKT/mTOR signaling cascades have a key role in regulating autophagy^41,42^. To explore the molecular mechanisms underlying the effects of ATO on autophagy, we analyzed the level of p-ERK1/2 (Thr202/Tyr204), ERK1/2, p-AKT (Ser473), AKT, p-mTOR (Ser2448) and mTOR after different exposure times (Fig. 6). ATO (2.5 μM) increased the ratio of p-ERK1/2/ERK1/2 in a time-dependent manner between 1 h and 8 h (Fig. 6A, B). In addition, ATO (2.5 μM) decreased the ratio of p-AKT/AKT between 4 h and 8 h, and time-dependently reduced the ratio of p-mTOR/mTOR between 1 h and 8 h (Fig. 6A, C, D). Furthermore, in macrophages treated with 2.5 μM ATO for 2–8 h, both the LC3II/LC3I ratio and p62 degradation were increased (Fig. 6A, E, F), suggesting that the inhibition of the PI3K/AKT/mTOR pathway and increased p-ERK1/2 were major characteristics of ATO-induced autophagy. To further support this conclusion, the macrophages were treated with inhibitors of class I PI3K (LY294002) or mTOR (rapamycin). LY294002 suppressed AKT and mTOR phosphorylation (Fig. 6G, I, J), whereas rapamycin inhibited mTOR phosphorylation, as compared to controls (Fig. 6G, J). LY294002 and rapamycin further increased the LC3II/I ratio with respect to the treatment with 2.5 μM ATO alone (Fig. 6K). In addition, NAC promoted AKT and mTOR phosphorylation, compared to controls (Fig. 6G, I, J), and inhibited LC3II conversion and p62 degradation (Fig. 6K, L). Remarkably, NAC prevented the changes induced by 2.5 μM ATO in AKT/mTOR signaling, as well as in the expression of autophagy-related proteins (Fig. 6G, I–L). These results indicated that ATO induces autophagy by inhibiting the PI3K/AKT/mTOR pathway in a ROS-dependent manner.

### ATO administration accelerates autophagy in the aorta of ApoE^−/−^ mice and reduces atherosclerotic lesions

**Fig. 7:**
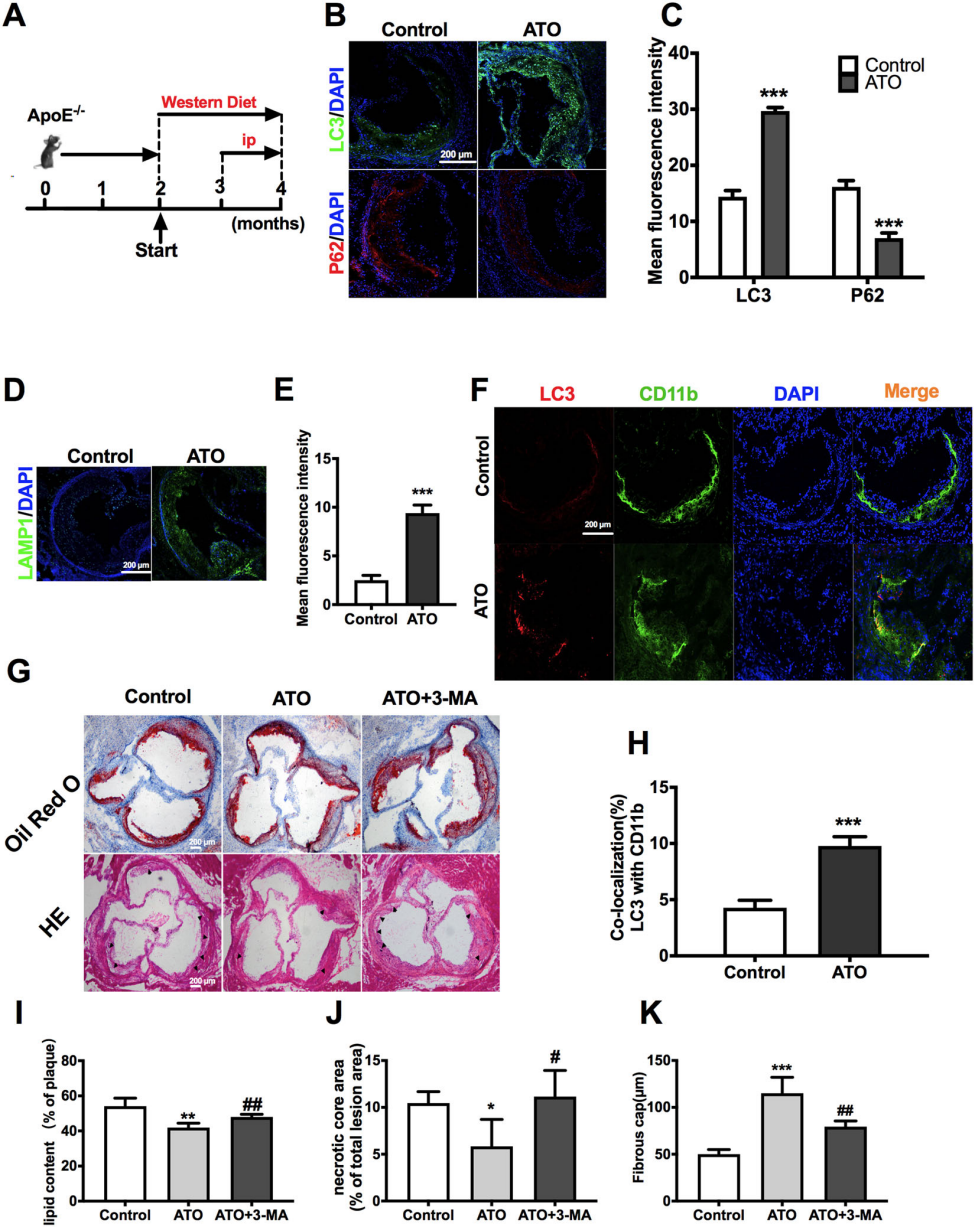
ATO administration accelerates autophagy in the aorta of ApoE^−/−^ mice and reduces atherosclerotic lesion. **A** ApoE^−/−^ mice were fed a western diet and were treated intraperitoneally with ATO (2.5 mg/kg) or the same volume of vehicle in saline as described in methods to establish early-stage atherogenesis lesions. **B**, **C** IF images of early-stage atherosclerotic (ApoE-KO) aortic roots stained with antibodies against LC3 (green), p62 (red) and DAPI (blue). Scale bars = 200 μm (*n* = 3 mice per group). **D**, **E** Representative immunofluorescence images of atherosclerotic aortic roots stained with antibodies against LAMP1 (green) and DAPI (blue). Scale bars = 200 μm (*n* = 3 mice per group). **F**, **H** Co-localization of LC3 (red) and CD11b (green) was also analyzed in the same aortic roots, and DAPI (blue) was added to label nuclei. Scale bars = 100 μm. **G** Representative Oil Red O and H&E staining of cross sections of aortic roots in the control, ATO, and ATO + 3-MA groups (*n* = 4 mice per group). The arrow indicates necrotic core. Scale bars = 200 μm. **I** The lipid content was calculated and analyzed from Oil Red O staining images. **J**, **K** The nerotic core area and thickness of fibrous cap were calculated and analyzed from H&E staining images. Data are expressed as mean ± SD from 3 independent experiments. *p vs. Control, #*p* vs. ATO. *p < 0.05, **p < 0.01, ***p < 0.001, ^#^*p* < 0.05, ^##^*p* < 0.01, and ns means non-significant.

The degradation of lipid droplets via the autophagy-lysosomal pathway is impaired in atherosclerosis^43^. ApoE^−/−^ mice were fed with a “western diet” for 2 months to establish an early atherosclerosis model. The ATO group was intraperitoneally administered with ATO in saline at a dose of 2.5 mg/kg every other day from the 12th to the 16th week (Fig. 7A). ATO increased the fluorescence intensity of LC3 and LAMP1, while decreased that of p62 in the aortic sinus, compared to controls (Fig. 7B–E). Furthermore, ATO enhanced LC3 fluorescence intensity in CD11b^+^ cells (Fig. 7F, H). These results indicated that macrophage displayed characteristic features of autophagy in the aorta of ATO-induced ApoE^−/−^ mice. Consistently, ATO promoted the autophagy-lysosomal pathway in THP-1 cells and even PBMCs in a dose- or time-dependent manner, as demonstrated by mRFP-GFP-LC3 staining and WB analysis (Supplementary Fig. S2).

To further verify that ATO-induced autophagy contributed to the reduction of atherosclerotic lesions, ATO (2.5 mg/kg) was combined with 3-MA (15 mg/kg) (Fig. 7A). Indeed, ATO reduced the ratio of necrotic core/plaque area and lipid accumulation, and increased the thickness of the fibrous cap, as compared to controls (Fig. 7G, I, J, K). However, all these ATO-induced changes were reversed by 3-MA treatment (Fig. 7G, I, J, K). These results collectively demonstrated that ATO reduced the atherosclerotic lesions in early AS by enhancing autophagy in macrophages. Moreover, we assessed viability of EC and SMC after treated with ATO (Supplementary Fig. S1a, b). The results showed that viability of EC and SMC were not decreased after exposure to ATO for 16–24 h at a concentration below 10 μM. Interestingly, when the SMC was incubated with supernatant of macrophages pre-treated with ATO (5 μM) for 2–8 h, the SMC viability was increased (Supplementary Fig. S1c). SMC can promote the formation of thick fibrous caps, thus stabilizing AS plaque^44^. Therefore, these data suggested that ATO may also alleviates AS plaque burden by promoting SMC survival.

## Discussion

**Fig. 8:**
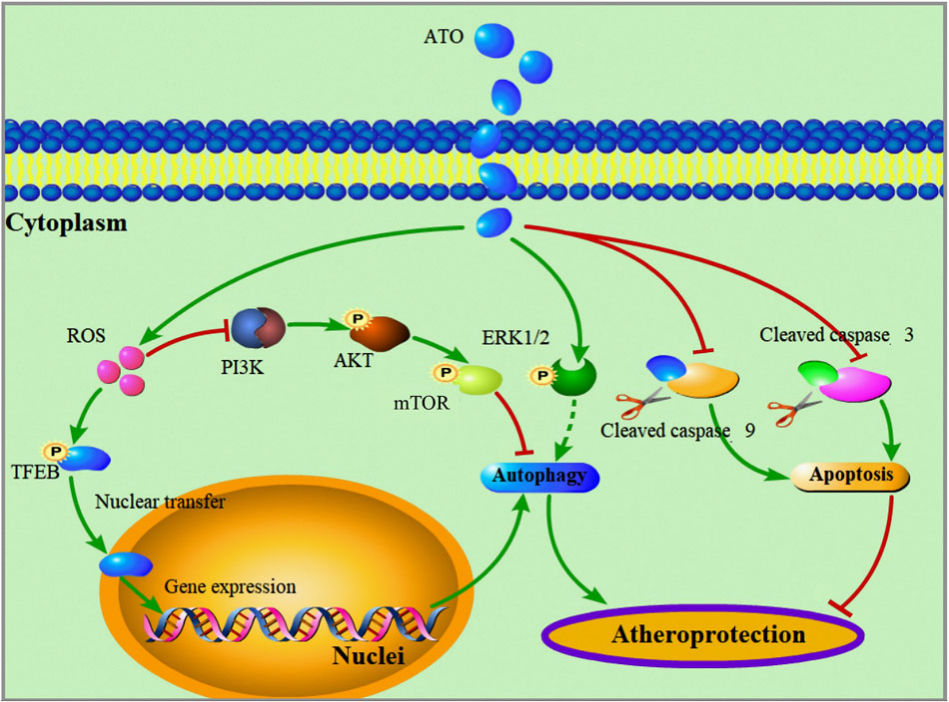
Proposed model describing the mechanism of autophagy activation and atheroprotection by ATO. The green arrows represent promotion. The red arrows with flat end represent inhibition.

In this study, we demonstrated that ATO induced autophagy in RAW264.7 cells, THP-1 cells, PBMCs, and macrophage-derived foam cells, meanwhile it reduced atherosclerotic lesions in vivo. Our findings provided insights into the possible mechanism underlying ATO-induced autophagy (Fig. 8), and suggested that ATO is a potential therapeutic option for atherosclerosis at early stages.

Autophagy is widely studied because of its relevance in cardiovascular diseases^45^. However, the therapeutic applications of ATO are limited due to its toxicity^46^. In this study, its ability to induce autophagy was achieved following an optimization of ATO doses in macrophages without affecting cell viability. The ATO-induced increase of LC3II/LC3I ratio and p62 degradation provided further support for autophagy induction. More importantly, LAMP1 labeling, as well as cell staining with LysoTracker and AO, demonstrated that ATO increased lysosomal activity, while the lysosomal inhibitor CQ blocked the ATO-induced degradation of p62. In addition, staining with mRFP-GFP-LC3 adenovirus confirmed that ATO promoted the fusion between autophagosomes and lysosomes. These results indicated that ATO promotes the completion of the autophagic flux.

An intricate crosstalk exists between autophagy and apoptosis^39^. Studies showed that macrophage autophagy inhibits apoptosis induced by lipid accumulation in atherosclerosis^47^. However, other studies indicated that autophagy promotes the elimination of damaged cells by apoptosis^48^. In this study, macrophages and foam cells triggered autophagy at 2 h after ATO treatment, earlier than the inhibited expression of apoptosis markers (i.e., cl-caspase-3 and cl-caspase-9) observed at 24 h. However, more apoptosis markers should be investigated to confirm that ATO inhibits apoptosis. Notably, there is a complex interaction between the apoptotic machinery (Bcl-2) and autophagy protein (Beclin 1)^49–51^. However, the precise mechanism of Beclin 1 mediated inhibition of apoptosis is not yet clear, and may be related to unregulated autophagy^52,53^. In our study, ATO increased Beclin-1 expression in macrophages. Whether ATO induces autophagy and inhibits apoptosis by affecting the interaction between Beclin-1 and Bcl-2 needs to be further explored.

ROS play an important role in the maintenance of homeostasis^54^, and have been used as inducers of cellular autophagy^55,56^. In this study, ROS were rapidly produced after a 1-h treatment with ATO, and disappeared after 2 h. Notably, after 2 h, ATO induced the activation of *Atgs*, the accumulation of LC3II, and the degradation of p62, suggesting that ROS generation preceded the initiation of autophagy. Further, NAC effectively blocked the autophagy activation by ATO. However, 3-MA failed to prevent ATO-induced ROS production. These data supported the notion that ROS accumulation exerts an upstream role in ATO-induced autophagy.

It is known that the suppression of PI3K/AKT/mTOR pathway may lead to cell survival or death via autophagy or apoptosis, respectively^57,58^. In our study, ATO was found to decrease the levels of p-AKT and p-mTOR in macrophages. Furthermore, ATO exhibited similar effects to those of the LY294002 and rapamycin, both of which also induced autophagy in macrophages. Overall, these observations indicated that the suppression of the PI3K/AKT/mTOR signaling pathway was a major characteristic of ATO-induced autophagy. In addition, NAC markedly reversed the drug-induced effects on the PI3K/AKT/mTOR cascade, as well as on the levels of autophagy-related proteins. These data suggested that, early ROS accumulation leads to autophagy via the inhibition of AKT/mTOR phosphorylation in ATO-treated macrophages. On the other hand, ERK1/2 activation has also been shown to promote autophagy^59,60^, and in our results, ATO could increase p-ERK1/2 levels in macrophages. However, it needs to be further explored whether ATO also triggers autophagy activation by activating ERK1/2.

TFEB increases the number and activity of lysosomes by regulating the transcription of target genes involved in lysosomal biosynthesis^61^. Our results indicated that ROS-dependent nuclear translocation of TFEB plays a crucial role in ATO-induced activation of the autophagy-lysosomal pathway. First, ATO enhanced the number of lysosomes and promoted the fusion between autophagosomes and lysosomes by inducing TFEB translocation to the nucleus. When TFEB was knocked down, lysosomal biogenesis and autophagy were impaired in ATO-treated macrophages. Next, in the presence of NAC, ATO failed to promote TFEB nuclear translocation, as well as to upregulate autophagy- and lysosome-related proteins. Furthermore, in this study, we also observed that p-mTOR and p-ERK1/2 were regulated by ATO, both of which are the main kinases that promote TFEB nuclear translocation. Consistently, TFEB siRNA did not inhibit ATO-induced ERK1/2 phosphorylation. However, recent studies have shown that ROS promotes the nuclear translocation of TFEB by directly oxidizing the cysteine residue (mTOR-independent) through a short-term action^39,62^. In our study, ATO can promote a rapid nuclear translocation of TFEB (15 min), which is too fast for an mTOR-based pathway. Therefore, we propose that the oxidation of the sole cysteine residue C211 under ATO treatment is responsible for the nuclear translocation of TFEB. Consistently, our results showed that after a 15-min ATO treatment, wild-type TFEB was rapidly translocated into the nucleus. In contrast, the rapid translocation of TFEB C211A was not observed (Fig. 4J, K). However, whether ATO-induced nuclear translocation of TFEB depends on the mTOR or ERK1/2 pathway should be clarified.

In atherosclerosis, lipid accumulation in the lysosomes leads to progressive lysosomal dysfunction and blockade of the autophagic flux^63^. Studies have pointed out that a variety of herbal medicines can accelerate the outflow of cholesterol by promoting autophagy in macrophages^64,65^. However, studies on drugs that can decelerate AS by promoting autophagy are very limited. In our study, ATO promoted the autophagy-lysosomal pathway in both THP-1 cells and PBMCs in vitro. More importantly, ATO promoted autophagy in the aorta of ApoE^−/−^ mice and reduced the atherosclerotic lesions in early AS. Further, these therapeutic effects of ATO were reversed by 3-MA treatment. We have explored the safe dose of ATO in vivo in another study (data not shown). We also proved that macrophages incubated with ATO produce substances promoting SMC survival in the supernatant. It may also be a reason for promoting plaque stability.

In conclusion, we demonstrated that ATO induces autophagy through multiple mechanisms and inhibits the expression of apoptosis markers in macrophages. Here, we provide a new perspective for the treatment of atherosclerosis at early stage. It is anticipated that further investigation of ATO effects in pre-clinical and clinical settings will result in the development of a new effective therapy for atherosclerosis.

### Ethics declarations

The experimental procedures and study design were conducted in accordance with institutional guides for animal experiments approved by the Experimental Center of the Second Affiliated Hospital of Harbin Medical University.

## Supplementary information

Analysis of efficiency of TFEB silence, expression of p-ERK and cell viability of EC and SMC.

ATO promotes autophagy in THP-1 cells and PBMCs.

Supplementary Figure Legends

## Data Availability

Data are available from the corresponding author upon request.
