# Supplementary figures and images for: Arsenic trioxide induces macrophage autophagy and atheroprotection by regulating ROS-dependent TFEB nuclear translocation and AKT/mTOR pathway
Source: Cell Death Dis. 2021 Jan 18;12(1):88. doi: 10.1038/s41419-020-03357-1 (PMC7814005; doi:10.1038/s41419-020-03357-1)

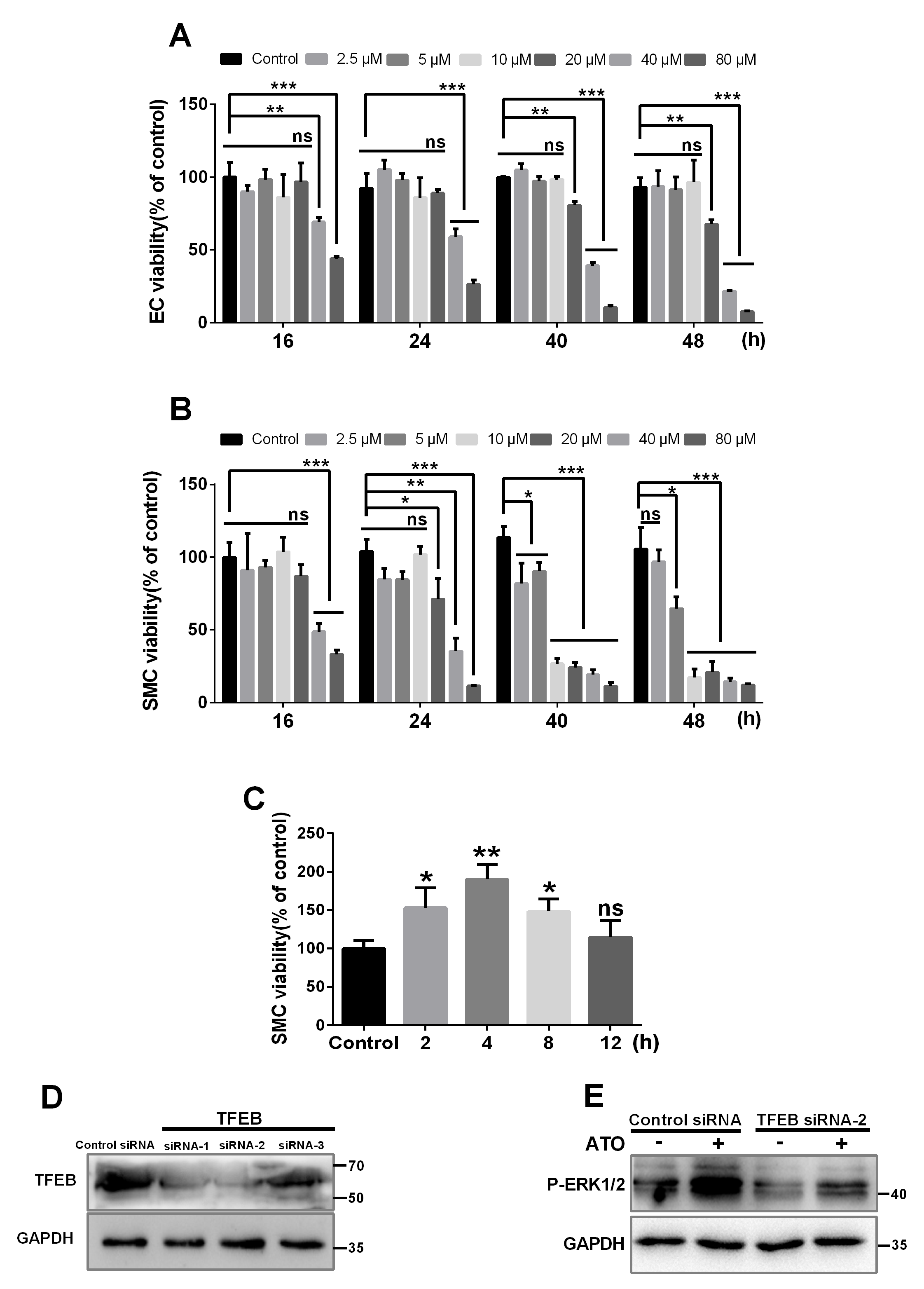

Supplement: Supplementary file 1 — Analysis of efficiency of TFEB silence, expression of p-ERK and cell viability of EC and SMC. [file 41419_2020_3357_MOESM1_ESM.tif]

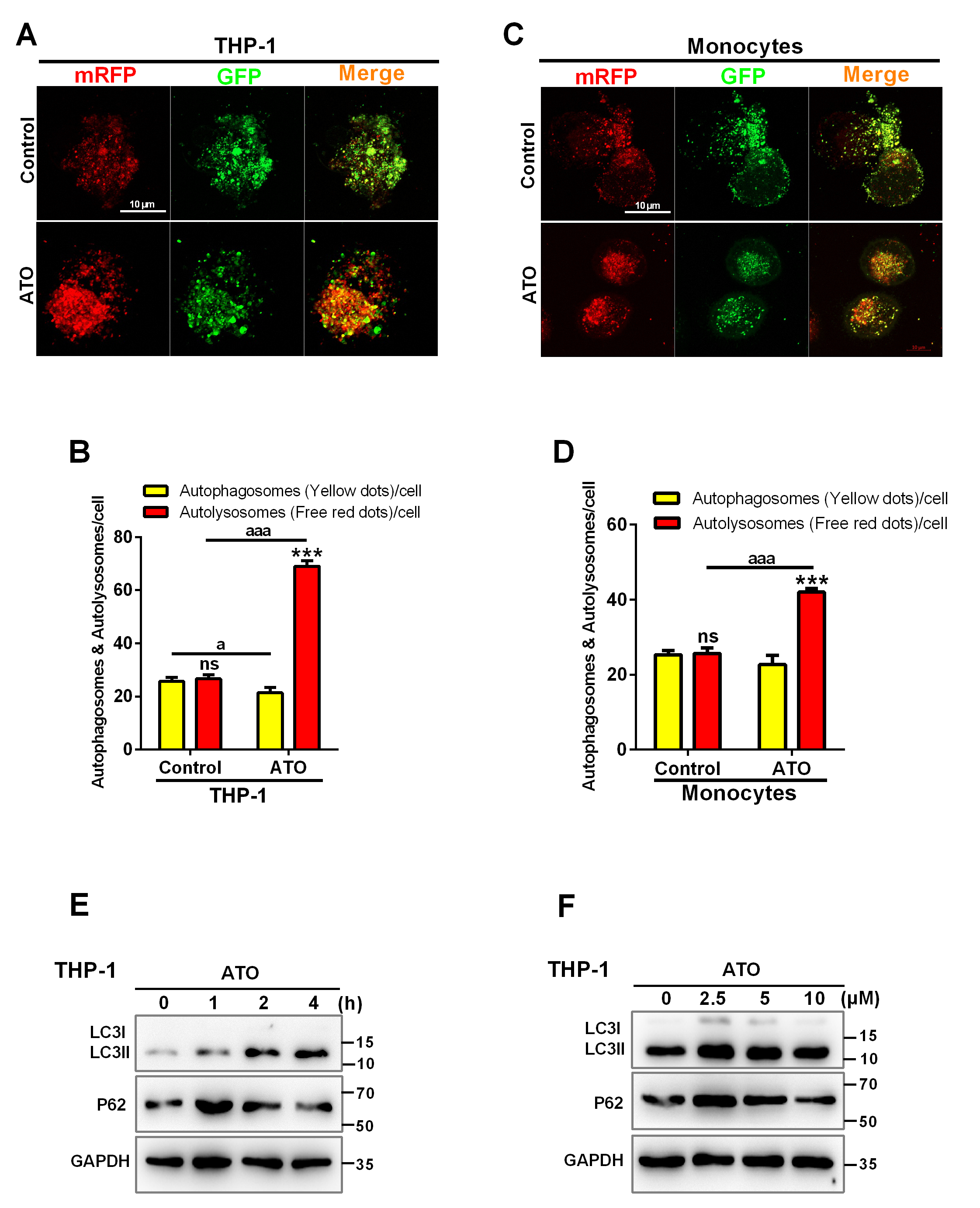

Supplement: Supplementary file 2 — ATO promotes autophagy in THP-1 cells and PBMCs. [file 41419_2020_3357_MOESM2_ESM.tif]
