# Supplementary material for: Arsenic trioxide induces macrophage autophagy and atheroprotection by regulating ROS-dependent TFEB nuclear translocation and AKT/mTOR pathway
Source: Cell Death Dis. 2021 Jan 18;12(1):88. doi: 10.1038/s41419-020-03357-1 (PMC7814005; doi:10.1038/s41419-020-03357-1)
Supplement: Supplementary file 3 — Supplementary Figure Legends [file 41419_2020_3357_MOESM3_ESM.doc]

**Fig. S1**

**Analysis of efficiency of TFEB silence, expression of p-ERK and cell viability of EC and SMC.**

(**a**, **b**) EC and SMC were treated with ATO at indicated concentrations and time points, cell viability was measured by the MTT assay. (**c**) RAW264.7 was pre-treated with 5 μm ATO for 0, 2, 4, 8, and 12 hours, and the supernatant was collected and incubated with SMC for 12 hours. cell viability of SMC was measured by the MTT assay. (**d**) RAW264.7 were transfected with negative control siRNA or three TFEB siRNAs, the efficiency of silence was analysed by WB. (**e**) WB analysis of TFEB effect on the expression of p-ERK1/2 in RAW264.7 cells as assessed autophagy specific proteins following indicated treatments. *p < 0.05, **p < 0.01, ***p < 0.001, and ns means non-significant.

**Fig. S2**

**ATO promotes autophagy in THP-1 cells and PBMCs.** (**a**-**d**) THP-1 and PBMCs were transiently transfected with GFPmRFP-LC3 for 48 h and subsequently treated with ATO (2.5 μM) for 2 h. Cells were observed by confocal microscope, and the colocalization of green (GFP-LC3) and red (autophagosome-lysosome) fluorescence was analysed. Scale bars=10 μm. (**e**) WB analysis of LC3II/LC3I and p62 expression in THP-1 cell after treated with 2.5 μM ATO for 1, 2, 4 h. (**f**) WB analysis of LC3II/LC3I and p62 in THP-1 cell after treated with ATO (2.5, 5, 10 μM) for 2 h. ****p*<0.001 vs. control, aaa*p*< 0.001 vs. Specified group, and ns means non-significant.
